# Novel members of the AGAMOUS LIKE 6 subfamily of MIKC^C^-type MADS-box genes in soybean

**DOI:** 10.1186/1471-2229-13-105

**Published:** 2013-07-20

**Authors:** Chui E Wong, Mohan B Singh, Prem L Bhalla

**Affiliations:** 1 Plant Molecular Biology and Biotechnology Laboratory, ARC Centre of Excellence for Integrative Legume Research, Melbourne School of Land and Environment, The University of Melbourne, Parkville, Victoria 3010, Australia

**Keywords:** Development, Floral meristem, MADS-box transcription factor, Soybean

## Abstract

**Background:**

The classical (^C^) MIKC-type MADS-box transcription factors comprise one gene family that plays diverse roles in the flowering process ranging from floral initiation to the development of floral organs. Despite their importance in regulating developmental processes that impact crop yield, they remain largely unexplored in the major legume oilseed crop, soybean.

**Results:**

We identified 57 MIKC^c^-type transcription factors from soybean and determined the *in silico* gene expression profiles of the soybean MIKC^c^-type genes across different tissues. Our study implicates three MIKC^c^-type transcription factors as novel members of the *AGAMOUS LIKE 6* (AGL6) subfamily of the MIKC^C^-type MADS-box genes, and we named this sister clade PsMADS3. While similar genes were identified in other legume species, poplar and grape, no such gene is represented in *Arabidopsis thaliana* or rice. RT-PCR analysis on these three soybean PsMADS3 genes during early floral initiation processes revealed their temporal expression similar to that of *APETALA1*, a gene known to function as a floral meristem identity gene. However, RNA *in situ* hybridisation showed that their spatial expression patterns are markedly different from those of *APETALA1.*

**Conclusion:**

Legume flower development system differs from that in the model plant, Arabidopsis. There is an overlap in the initiation of different floral whorls in soybean, and inflorescent meristems can revert to leaf production depending on the environmental conditions. MIKC^C^-type MADS-box genes have been shown to play key regulatory roles in different stages of flower development. We identified members of the PsMADS3 sub-clade in legumes that show differential spatial expression during floral initiation, indicating their potential novel roles in the floral initiation process. The results from this study will contribute to a better understanding of legume-specific floral developmental processes.

## Background

Flower development in plants involves tightly regulated processes starting from floral initiation to flower formation. The underlying processes have been extensively investigated, as flower development is an important agronomic trait that determines crop yield. Various transcription factors are essential in regulating these developmental processes, including the family of MADS-box transcription factors.

The MADS-box transcription factors, especially the plant-specific classical (^C^) MIKC-type MADS-box genes, are known to play key regulatory roles in different stages of flower development. Their roles in coordinating floral developmental processes have been revealed by functional studies largely carried out in the model plant, *Arabidopsis thaliana*. The MIKC^C^-type genes are characterised by a conserved structural organisation of the **M**ADS-box, **I**ntervening-, **K**eratin-like- and **C**-domains. The highly conserved MADS-domain and the weakly conserved I-domain are required for DNA binding, while the strongly conserved K-domain and the variable C-domain regulate protein interactions [1].

Genome-wide analyses of the MIKC^C^-type genes have been carried out in Arabidopsis [2], rice [3] and poplar [4]. While Arabidopsis and rice genomes have similar numbers of MIKC^C^-type genes (39 vs. 38), poplar has 55 of these genes, suggesting a higher birth rate compared to Arabidopsis or rice. These MIKC^c^-type genes can be divided into 15 distinct gene clades with each clade named after the first member identified [3,5]. All but two (TM8 and OsMADS32) are found in Arabidopsis [3,5], and the FLC clade may be absent from the rice genome [3]. It remains unclear whether all clades are present in the poplar genome, as no TM8 genes were used in the phylogenetic analysis [4].

The SQUA subfamily clade includes four Arabidopsis members, APETALA1 (AP1), CAULIFLOWER (CAL), FRUITFULL (FUL) and AGAMOUS-LIKE 79 (AGL79) [2]. The functions of AP1, CAL and FUL have been characterised, indicating that they play partially redundant roles in determining floral meristem identity [6]. The SEPALLATA (SEP) family belongs to the AGL2 clade, and there are four members documented in Arabidopsis [7]. All four members (SEP1, SEP2, SEP3 and SEP4) play redundant functions in determining floral organ identity and floral meristem determinacy. AP1 has been shown to bind directly to the SEP3 promoter, hence increasing the expression of *SEP3* rapidly [8]. The AGL6 subfamily has a relatively small representation (only two genes, AGL6 and its paralog AGL13) in Arabidopsis. While no knockout phenotype has been described for either of these genes in Arabidopsis, studies in rice, maize and *Petunia hybrida* have largely demonstrated the roles of the AGL6 subfamily in regulating floral organ identity and floral meristem determinacy, indicating redundant roles with closely related genes including *SEP*[9-11]. A phylogenetic study showed that subfamilies of SQUA, SEP and AGL6 are always rooted together in one superclade, which may be correlated with their overlapping roles in regulating flower development.

A total of 212 MADS-box genes were predicted in the recent genome sequence of soybean [12]. Earlier we reported the diversification of some gene expression and microRNAs in legume SAM [13-16]. However, much remains to be learned about these genes, especially given their potential impact on crop production. Soybean is the largest legume crop in the world and accounts for greater than 50% of the global oilseed production. In this study, we identified all the soybean MIKC^c^-type MADS-box genes using the current Glyma1.0 gene set and identified potential phylogenetic relationships to their Arabidopsis, rice and poplar counterparts. We examined the expression patterns across different soybean tissues for the entire family. Intriguingly, the results revealed a novel AGL6 sister clade of MIKC^c^-type genes in soybean, and we focused our subsequent analysis on members of this novel sub-clade.

## Results and discussion

### Molecular evolutionary analysis of soybean MIKC^c^-type MADS-box transcription factors

When we searched the soybean predicted gene set available at Phytozome [12] for sequences containing both a MADS-box and K-domain, we identified a total of 57 sequences. Subsequent inspection revealed three of the sequences were incomplete. We attempted to obtain a full-length sequence for these genes using gene prediction software on the genome sequence surrounding these partial sequences but did not yield any results. Therefore, we omitted these sequences from further analysis. To investigate their phylogenetic relationships with MIKC^c^-type genes from Arabidopsis, rice and poplar, reported MIKC^c^ group protein sequences [3-5] were retrieved from their respective databases. A total of 159 conceptually translated protein sequences were used in the phylogenetic analysis.

**Figure 1:**
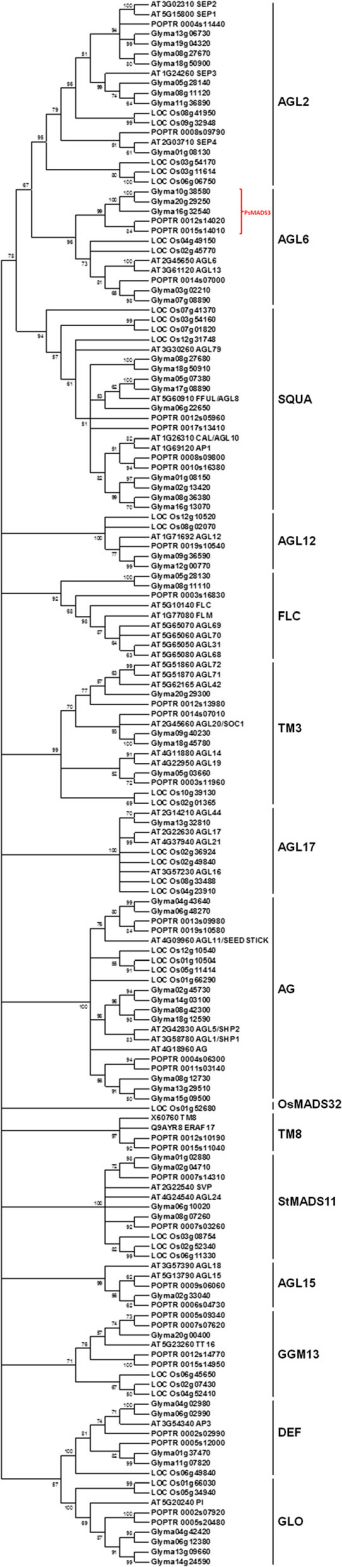
**Relationships among representative members of MIKC**^**c**^**-type genes.** a. The phylogenetic tree was based on MUSCLE alignments of conceptual protein sequences spanning the MADS-, I- and K-domains of sequences from Arabidopsis, rice, poplar and soybean. The unrooted bootstrap consensus tree was constructed using the Maximum Likelihood method implemented in Mega 5. The number for each node is the bootstrap percentages (200 replications), and nodes with less than 50% bootstrap values were collapsed. Fifteen clades of MIKC^c^-type genes are indicated, with PsMADS3 potentially representing a novel sister clade of AGL6.

Fifteen existing clades were identified from the generated phylogenetic tree: AGL2, AGL6, SQUA, AGL12, FLC, TM3, AGL17, AG, OsMADS32, TM8, STMADS11, AGL15, GGM13, DEF, and GLO (Figure 1). The relationships among the different clades are similar to other reports [2-5]. With the exception of OsMADS32 and TM8, soybean genes are found in all clades, and the number of soybean sequences within each clade varies from one (AGL15 and AGL17) to nine (SQUA1), with most genes occurring in duplicate. Consistent with a previous report [5], the genes in AGL2, SQUA and AGL6 form a superclade together (Figure 1). Within the AGL6 clade, a strongly supported internal branch seems to be separate from the existing AGL6 members, suggesting it is a novel sister clade not represented in Arabidopsis or rice. This novel sub-clade consists of three soybean genes, Glyma16g32540 and a homeolog pair, Glyma10g38580 (GmMADS3a) and Glyma20g29250 (GmMADS3b). The top BLASTX match of these genes is a MADS-box transcription factor from garden pea, which is annotated as PsMADS3 [17] (data not shown). Therefore, we named it the PsMADS3 sub-clade.

**Figure 2:**
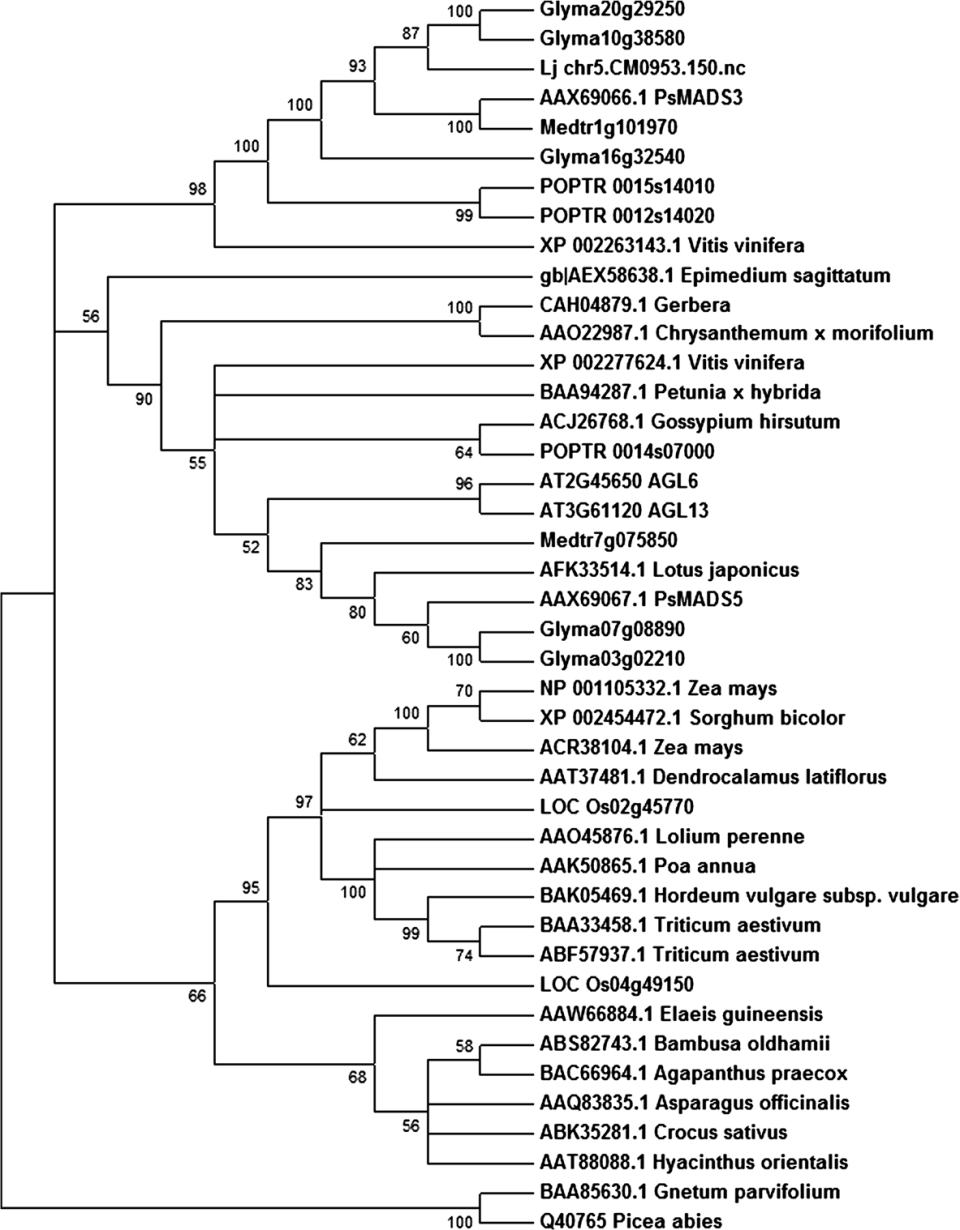
**Phylogeny of *AGL6 *genes.** The tree was produced as in Figure 1 but from complete protein sequences. The predicted peptide sequence of GmMADS3a (Glyma10g38560) was used for a BLASTP search at NCBI. The top 40 matches (E value < 1e-50) were downloaded from NCBI and filtered for duplicated or short sequences prior to phylogenetic analysis. The tree was rooted by using the gymnosperm AGL6 as the outgroup. Plants used for analysis: *Agapanthus praecox, Arabidopsis thaliana, Asparagus officinalis, Bambusa oldhamii, Chrysanthemum morifolium, Crocus sativus, Dendrocalamus latiflorus, Elaeis guineensis, Epimedium sagittatum, Gerbera jamesonii, Glycine max, Gnetum parvifolium, Gossypium hirsutum, Hordeum vulgare, Hyacinthus orientalis, Lolium perenne, Lotus japonicus, Medicago truncatula, Oryza sativa, Petunia hybrida, Picea abies, Pisum sativum, Poa annua, Populus trichocarpa, Sorghum bicolor, Triticum aestivum, Vitis vinifera, Zea mays.*

We next examined whether similar orthologous members of PsMADS3 exist in species other than soybean, garden pea and poplar. A BLASTP search at the NCBI public database identified potential PsMADS3 sequences from other species (data not shown). Using the top matching 40 orthologs including sequences from the sister clade, AGL6, for phylogenetic analysis, three distinct groups were identified in the tree rooted with gymnosperm AGL6 as the outgroup (Figure 2). One clade groups all of the monocot sequences, whereas the other two clades contain AGL6 and PsMADS3 sequences (Figure 2). As we were only using sequences available in the public database, it is likely that similar PsMADS3 sequences exist in species other than those examined. Future studies with increased taxon sampling will help to clarify the node. Members of the PsMADS3 sub-clade are represented in not only legume species but also poplar and grape (Figure 2). As the genome sequences for Arabidopsis and rice are available and well annotated, we are confident that members of this PsMADS3 sub-clade are absent from these two species. Furthermore, the observation that no orthologous PsMADS3 genes is found among the top matching sequences from other monocots including wheat and maize (Figure 2) implies that such genes may be absent from monocot. The PsMADS3 clade may have evolved after monocot-dicot divergence and following the emergence of the Arabidopsis species. These genes may have been overlooked previously due to their absence in Arabidopsis and rice. In fact, none of these legume genes were analysed in a recent study on eudicot AGL6 [18].

### In silico analysis of soybean MIKC^c^ gene expression patterns

**Figure 3:**
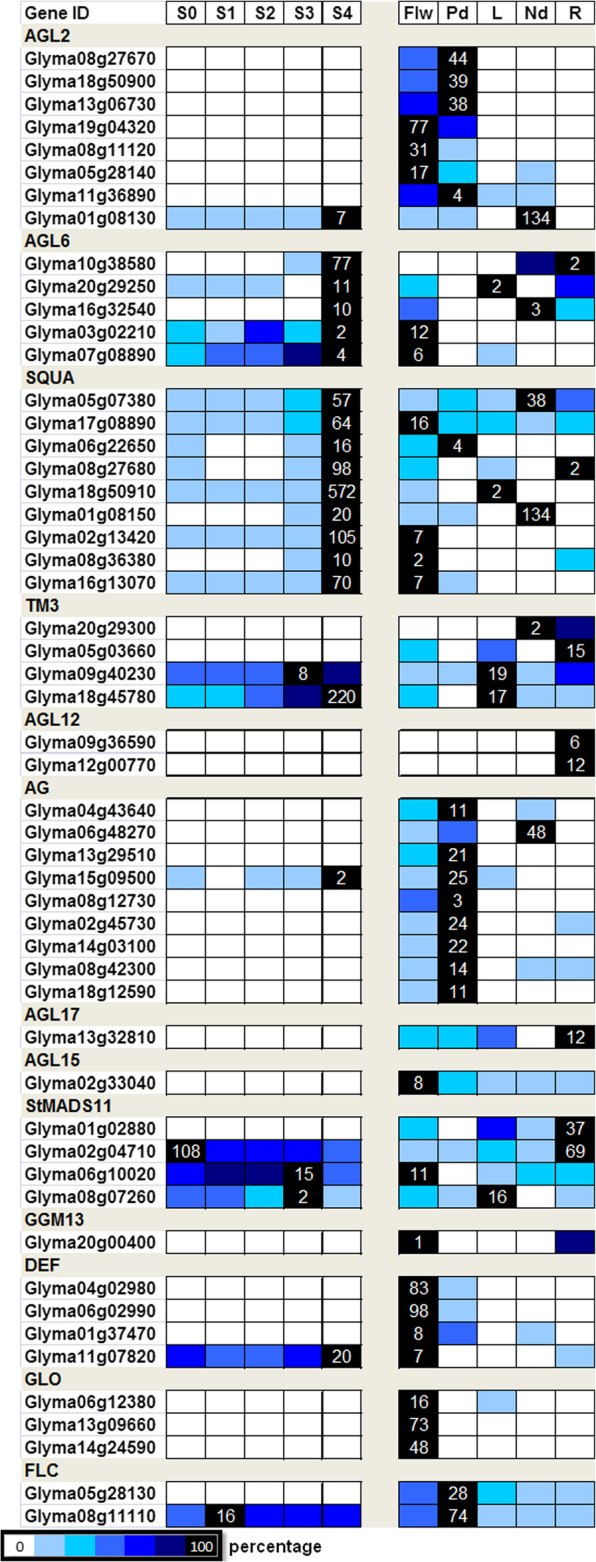
**Expression profiles of soybean MIKC**^**c**^**-type transcription factors.** The highest expression level for each gene across two different sets of samples is given as an RPKM value (see Methods). The level of expression for a gene is represented as the percentage of the maximum expression level and colour coded from 0% (white) to 100% (black). S0-S4: samples derived from SAM after 10-day-old soybean plants were shifted to short-day growth conditions as described in the Methods at intervals of 0 short-day (S0), 1 short-day (S1), 2 short-day (S2), 3 short-day (S3) or 4 short-day (S4). Flw, flower; Pd, pod; L, leaf; Nd, nodule; R, root. AG-AGAMOUS, AGL2/6/12/15/17- AGAMOUS-LIKE2/6/12/15/17, SQUA-SQUAMOSA, FLC-FLOWERING LOCUS C, GGM13-*Gnetum gnemon* MADS box transcription factor13, StMADS11- *Solanum tuberosum* MADS11, TM3- Tomato MADS box transcription factor3, DEF:DEFICIENS, GLO:GLOBOSA.

We previously performed high-throughput RNA-sequencing on micro-dissected shoot apical meristems (SAMs) undergoing the early floral initiation process [29]. The samples were derived from soybean plants (10-day-old) subjected to different lengths of short-day (SD) treatments (0SD, 1SD, 2SD, 3SD and 4SD), and the induction of the floral meristem identity genes including *GmAP1* occurred on 4SD. Because genes that are involved in later processes of flowering such as floral organ development or only expressed in other tissues such as roots may not be captured by our dataset, we also used the transcriptome data reported by Libault and co-workers [19] to include a diverse range of tissues including flower, seed pod, root, nodule and root tip in our *in silico* analysis (Figure 3).

All identified soybean MIKC^c^-genes are expressed in at least one of the three reproductive tissues represented (reproductive SAM, flower and pod), except for members of the AGL12 clade, which are only expressed in the root (Figure 3). Arabidopsis *AGL12* is preferentially expressed in the root, and recent loss-of-function analyses have revealed its roles in not only regulating root meristem cell proliferation but also flowering transition [20,21]. Based on the soybean *AGL12-LIKE* expression profile, it is tempting to speculate that their functions in floral regulation may have been lost. A similar expression pattern was observed for most MIKC^c^-genes clustered within a clade. All duplicated genes are transcribed and have comparable expression profiles, especially in the reproductive SAM (Figure 3), suggesting their functional significance.

As for the superclade consisting of AGL2, SQUA, and AGL6, there are some notable differences in the gene expression profiles among the three clades (Figure 3). For example, all *AGL2-LIKE* genes except one (Glyma01g08130) are absent from the SAM during the early floral transition process but are expressed later in the floral developmental process in the flower and pod. This pattern is expected as these genes are known to be activated following *AP1* induction in Arabidopsis [22]. The phylogenetic tree indicates that Glyma01g08130 is the counterpart for Arabidopsis *SEP4*. In addition to being found in flower and pod like the rest of the *AGL2-LIKE* genes, it is also expressed in the SAMs during the floral initiation process and very highly in nodules. This pattern implies a likely diverged function of *GmSEP4* with additional roles in the early floral initiation process as well as in nodule formation. Glyma01g08150, one of the four soybean counterparts of Arabidopsis AP1, also likely plays a role in nodule formation. Although the expression of Glyma01g08150 is drastically induced on 4SD in the SAM (20 RPKM), the level of expression is 6-fold less than that in the nodule (134 RPKM; Figure 3). Intriguingly, its homeolog Glyma02g13420 is not expressed in the nodule but rather has the highest expression in the reproductive SAM (105 RPKM; Figure 1 &3), suggesting a functional divergence between this homeolog pair.

Although members of soybean AGL6 genes are expressed in the reproductive SAMs, changes in their transcript levels are not comparable with those of *PsMADS3-LIKE* and *SQUA-LIKE* genes during the early floral transition process, suggesting that the latter two clades are likely to play more prominent roles in the developmental transition process. Because there is no information available for *PsMADS3-LIKE* genes, we focused our study on members of this novel sister clade.

### Expression of GmMADS3 during the floral initiation process

**Figure 4:**
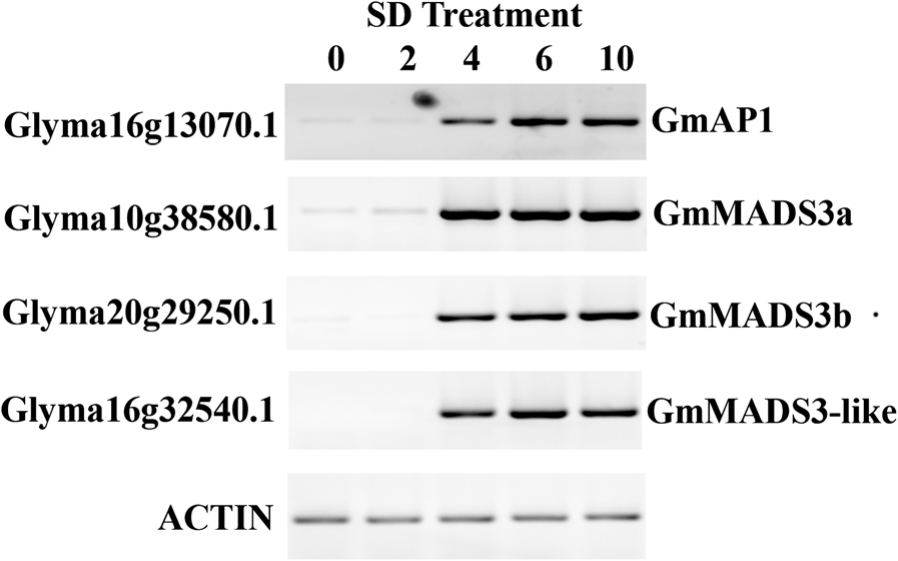
**RT-PCR analyses of soybean *PsMADS3-LIKE *and *GmAP1 *sequences during early floral initiation processes.**

We carried out RT-PCR analysis to verify the expression of this novel family in the soybean SAM during the early floral initiation process (Figure 4). RNA was extracted from dissected SAMs of plants undergoing 0, 2, 4, 6 and 10 SD treatments. RT-PCR was carried out with primers specific to each of the soybean members in this PsMADS3 branch (Glyma16g32540, Glyma10g38580, and Glyma20g29250) as well as to *GmAP1* (Glyma16g13070) as the control for the floral induction process. Consistent with our previous study [23], the induction of *GmAP1* occurs after the 4 short-day treatment. All three soybean genes in the PsMADS3 clade displayed a similar expression pattern to that of *GmAP1*, consistent with the *in silico* analysis.

**Figure 5:**
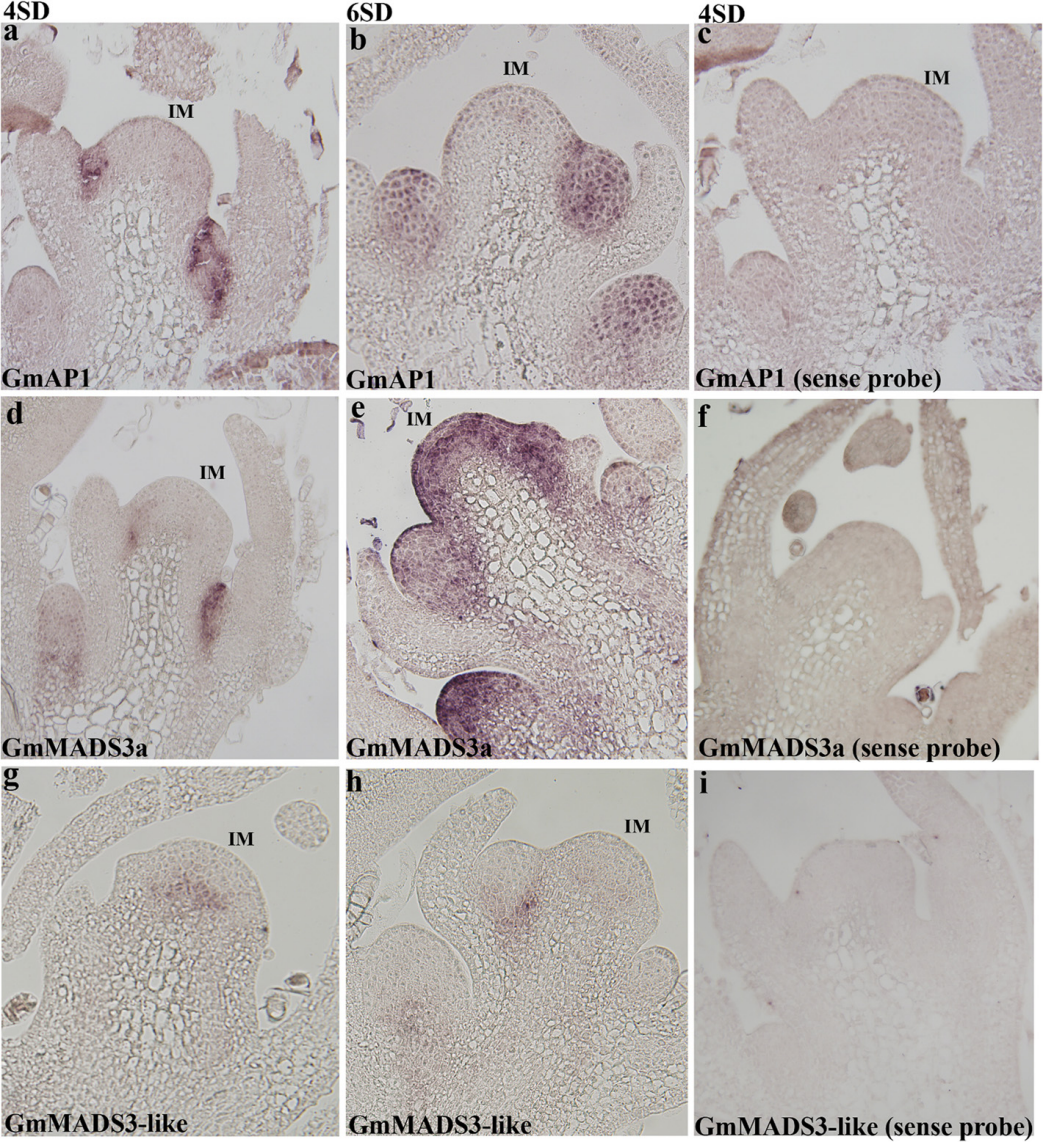
**Spatial expression pattern of soybean MIKC**^**c**^**-type transcription factors. a** &**b**. *GmAP1* (Glyma16g13070) expression was first detected in the incipient floral primordia of the inflorescence meristem on 4 SD, which was then expressed throughout the newly established floral meristem on 6SD. **d** &**e**. *GmMADS3* (Glyma10g38580, Glyma20g29250) was expressed in the incipient floral primordia of the inflorescence meristem and subsequently in the entire inflorescence meristem as well as the newly established floral meristems on 6SD. **g** &**h**. *GmMADS3-like* gene (Glyma16g32540) was detected in the centre of the inflorescence and floral meristems. **c, f** &**i.** Sections were hybridised with the sense probe of the corresponding gene, as indicated. IM: Inflorescence meristem.

To examine the spatial expression pattern of these genes during the floral initiation process, *in situ* hybridisation analysis was performed. On 4SD, *GmAP1* expression was detected in the incipient floral primordia of the inflorescence meristem (Figure 5a). On 6SD, newly established floral meristems became more prominent and *GmAP1* expression was detected throughout these meristems (Figure 5b). *GmMADS3a* (Glyma10g38580) exhibited a rather similar expression pattern to *GmAP1* on 4SD (Figure 5d), but its expression subsequently spread to the entire inflorescence meristem as well as to the newly established floral meristems on 6SD (Figure 5e). As the *GmMADS3* homeolog pair is almost identical in nucleotide sequence including the UTR regions (data not shown), no gene-specific probe could be made, and thus the signals observed may correspond to the expression of both genes. Nevertheless, the expression of *GmMADS3* throughout the entire inflorescence and floral meristem suggests that it can serve as both an inflorescence and floral meristem identity gene. Because the expression of *GmMADS3* initially overlaps with that of *GmAP1*, it likely performs similar functions as *GmAP1*. Its subsequent widespread expression in the inflorescence meristem may ensure all vegetative activities at the SAM are replaced with the initiation of the floral meristem at the meristem flanks.

The expression of Glyma16g32540 is distinct from that of *GmAP1* and *GmMADS3*. A weak signal associated with its expression was detected in the centre of the inflorescence meristem (Figure 5g); on 6SD, its expression was also observed in the centre of the newly emerged floral meristem (Figure 5h). The expression of Glyma16g32540 in the centre of the meristem indicates its potential regulatory roles in orchestrating events in the inflorescence meristem. The spatial expression pattern of the soybean *PsMADS3-LIKE* genes supports that these genes are novel, as their expression differs markedly from the spatial expression of closely related family members such as *GmAP1* (this study) or Arabidopsis *AGL6*[24].

## Conclusions

In contrast to Arabidopsis where the initiation timing of floral whorls does not overlap, the legume soybean has a flower development system with overlapping whorls [25]. Furthermore, unlike Arabidopsis that usually cannot undergo flowering reversion [26], the soybean inflorescent meristem can revert to leaf production when the environmental growth conditions are switched from SD to LD [27]. Because the MIKC^C^-type MADS-box genes play key regulatory roles in different stages of flower development, it is conceivable that members of the PsMADS3 sub-clade identified in this study could contribute to developmental plasticity in cooperation with key floral regulators such as GmAP1 or GmFLC. Future studies aimed at defining the interacting partners of these genes will aid in our understanding of the floral transition process.

## Methods

### Sequence and phylogenetic analysis

Conceptually translated protein sequences were retrieved from public databases (Phytozome, Rice Genome Annotation Project, TAIR and LjGDB). For the initial identification of the soybean MIKC^C^-type MADS-box transcription factors, all annotated genes were screened for both the MADS-box domain (PFAM00319) and K-domain (PFAM01486). The results were then manually inspected and filtered for truncated protein sequences, resulting in a total of 54 sequences (Additional file 1: Table S1). Sequences were imported into MEGA version 5 software for subsequent phylogenetic and molecular evolutionary analyses [28]. MUSCLE alignments of protein sequences spanning the MADS-, I- and K-domains were carried out using the default settings in MEGA. After alignment, the evolutionary history was estimated using the Maximum Likelihood method based on the JTT matrix-based model as implemented in Mega 5 with bootstrap analysis set at 200 replicates.

For the expression profile analysis, two separate transcriptome sequencing data were used [19,29]. The abundance for each gene was normalised within each dataset and expressed in reads per kb per million (RPKM) and are provided in Additional file 1.

### Plant growth and RNA extraction

Soybean plants [Glycine max. (L) Merr. Cv. Bragg] were grown in a greenhouse located at the University of Melbourne, Victoria, Australia. To induce flowering, 10-day-old plants were shifted to a growth chamber maintained at a constant temperature of 25°C with a 10-hr day (150 μmol m^-2^ s^-1^) and 14-hr night (short-day). Shoot apical meristems (SAMs) were micro-dissected, as previously described [24]. Total RNA was extracted from dissected SAM (approximately 80 SAMs per extraction) using the Qiagen RNeasy Mini Kit (Qiagen, Victoria, Australia) with on-column DNAse digestion.

### RT-PCR analysis

The Qiagen one-step RT-PCR kit was used according to the manufacturer’s instructions in all RT-PCR analyses. Total RNA (20 ng) isolated from the SAMs of 10-day-old soybean seedlings (0 SD) and from the SAMs of plants subjected to different short-day treatments (2, 4 or 6 SD) was used as the template in a 10-μl reaction volume for 25 amplification cycles. Primers used are:

Glyma10g38580F CAACTGGATAGAACGCTTGCACAAG

Glyma10g38580R CATCAATGGACGCTTAACGTACTATATAGC

Glyma16g32540F CTTGAGCTGACACAAAGGCA

Glyma16g32540R GCTTTGACTACCGTCTGTCTTG

Glyma20g29250F AGCTCGGAAGCACCTAACG

Glyma20g29250R CATCAATGGACCCTCAACTATAGC

Glyma16g13070F GCCTCAAAGAGCTTCAGAGTCTGGAGC

Glyma16g13070R AGAAAGCCTAGCCTTGTGACCA

ACTINF ATCATGTTTGAGACCTTCAATGTG

ACTINR CTCGAGTTCTTGCTCATAATCTAGG.

The soybean actin gene was used as an internal control. The PCR reactions were separated on 1% agarose gels containing 0.1 μg/μl ethidium bromide and visualised under UV light.

### RNA *in situ* hybridisation

The soybean shoot apices were dissected and fixed with 4% paraformaldehyde (Sigma, Victoria, Australia) in phosphate-buffered saline overnight at 4°C after vacuum infiltration. Subsequent fixation and hybridisation steps were followed as previously described [13].

## Competing interests

The authors declare that they have no competing interests.

## Authors’ contributions

CEW conducted all experiments and data analysis and wrote the manuscript. PB and MS designed and supervised the study and edited the manuscript. All authors read and approved the final manuscript.

## Acknowledgements

We wish to thank Professor Bernie Carroll for soybean seeds and Dr. Sheh May Tam and Dr. Lim Chee Liew for help with the phylogenetic analyses. Financial support from the Australian Research Council in the form of the ARC Centre of Excellence for Integrative Legume Research (CE0348212) andARC DP0988972 is also gratefully acknowledged.

## Supplementary Material

Additional file 1: Table S1 Expression levels of soybean MIKC^C^-type transcription factors in different tissues. Click here for file
